# Not Replaced, but Reinvented: AI Education Pathways to Prepare Future Physicians to Lead Healthcare Transformation

**DOI:** 10.5334/pme.2233

**Published:** 2025-11-21

**Authors:** Neil Mehta, Seysha Mehta, Alexandra Rubenstein, Sarah K. Wood

**Affiliations:** 1 Cleveland Clinic Lerner College of Medicine, Case Western Reserve University School of Medicine, Cleveland, Ohio, US; 2 Tufts University School of Medicine, Boston, MA, US; 3 Harvard Macy Institute, Harvard Medical School, Boston, MA, US

## Abstract

Artificial intelligence (AI) is rapidly transforming medical education and practice, challenging the cognitive role of physicians, and raising concerns about their future relevance. While this potential disruption sparks fear, it is also an unprecedented opportunity. Healthcare faces significant challenges — inequities, inefficiencies, and gaps in quality — that AI could help address. Instead of fearing change, physicians must take the lead in shaping the AI-integrated future of healthcare for the benefit of patients and society. Physicians, with their deep understanding of patient needs and systemic challenges, must lead this transformation — starting with medical education. The next generation of physicians should be prepared not just to work alongside AI, but to drive its development in ways that enhance patient outcomes and uphold medical ethics.

This Eye opener, authored by medical educators and students with diverse perspectives, proposes foundational skills for all physicians, including AI literacy, critical appraisal of AI-generated outputs, ethical considerations, and resilience in AI-independent decision-making. Beyond these core competencies, they outline specialized AI educational pathways — ranging from technical expertise to health system transformation and precision medicine — to prepare healthcare professionals for leadership in an AI-integrated future. Designing these curricula will require innovative collaboration with technological experts, data scientists, policymakers, clinicians, and educators. This educational reform demands systems thinking, interdisciplinary collaboration, faculty development, robust assessment frameworks, global perspectives, and a commitment to lifelong learning. Delaying this work risks ceding control of AI’s role in medicine to entities that may not always prioritize patient welfare. The future of AI-integrated healthcare will be shaped by those who take initiative. Medical educators must guide and train students to lead AI’s transformative potential, ensuring that technological advancements are leveraged for the benefit of patients and society. The time to act is now.

*“If we’re in denial — or if we’re simply not paying attention — we could lose the chance to shape this technology when it matters most.” – Kevin Roose NY Times 3/14/2025*

The accelerating advance of generative artificial intelligence is no longer a distant forecast; it is a present-day reality. Large language models (LLMs) are demonstrating remarkable capabilities in clinical data interpretation, differential diagnosis, and even complex clinical reasoning — tasks long considered the exclusive domain of physicians [1,2]. This sudden proficiency has sparked significant concern and anxiety among medical professionals about their future roles in an AI-integrated healthcare system [3,4,5].

While the apprehension is natural, this moment of disruption is not a threat, but rather a profound opportunity. To fear AI is to miss the point; we should instead fear the persistence of a healthcare system that is fundamentally struggling.

Let us be candid about the state of modern healthcare. It is beset by systemic problems: a crippling shortage of clinicians, epidemic burnout, and training pathways that are prohibitively long and expensive. For patients, the system is often inaccessible, inequitable, and inefficient, delivering inconsistent quality at an unsustainable cost. Generative AI, if developed and implemented thoughtfully, holds the potential to directly address many of these deep-seated issues [6,7,8,9]. It can automate administrative burdens, democratize medical knowledge, accelerate research, and personalize patient care at a scale never before possible.

However, the history of technology in medicine serves as a cautionary tale. It is rife with examples of physicians remaining on the sidelines during the design, adoption, and implementation of new tools. Burnout-inducing electronic health records (EHRs) are a painful testament to what happens when technology is done *to* clinicians rather than *with* them. This resulted in lost opportunities, frustrated providers, and a failure to realize digitization’s true potential [10,11].

We cannot afford to repeat this mistake with AI, a technology far more powerful and transformative than EHRs. By taking vocal leadership roles in the integration of AI, physicians can steer this revolution toward better outcomes for both patients and providers. We can help ensure these tools are built on sound clinical data, validated for safety and equity, and designed to augment clinicians—freeing them to focus on the human aspects of care that AI cannot replace: empathy, judgement, and the therapeutic relationship. Leadership is essential to uphold the very commitments that define responsible AI in medicine, such as those articulated by the National Academy of Medicine [12]. Principles like “advancing humanity,” “ensuring equity,” and “improving workforce well-being” are not technical specifications but deep clinical and ethical mandates. Without physicians at the helm to provide clinical context and prioritize ethical considerations, these goals risk becoming empty promises. It is this clinician-led guidance that will ensure AI tools are not just powerful, but also safe, fair, and genuinely beneficial in practice.

Instead of fearing disruption, physicians and trainees must prepare to meet this challenge. To do so, the first and most critical step is to fundamentally reform our system of medical education. The curriculum that trained physicians for the 20th century is insufficient for the 21st.

## Educational Pathways for AI Integration

To prepare the healthcare workforce for an AI-driven future, education must span the entire learning continuum—from undergraduate (UME) and graduate medical education (GME) to continuing medical education (CME). These pathways should equip healthcare professionals with the knowledge, skills, and ethical grounding essential to lead the shift toward more effective, AI-integrated systems. The current route to clinical expertise is long and requires mastery of extensive information — much of which AI can now help to manage. Training physicians to harness these tools will not only make them indispensable in an AI-enabled healthcare landscape, but, more importantly, empower those who best understand patient care and the system’s complexities to shape transformation that truly benefits patients and communities.

**Figure 1:**
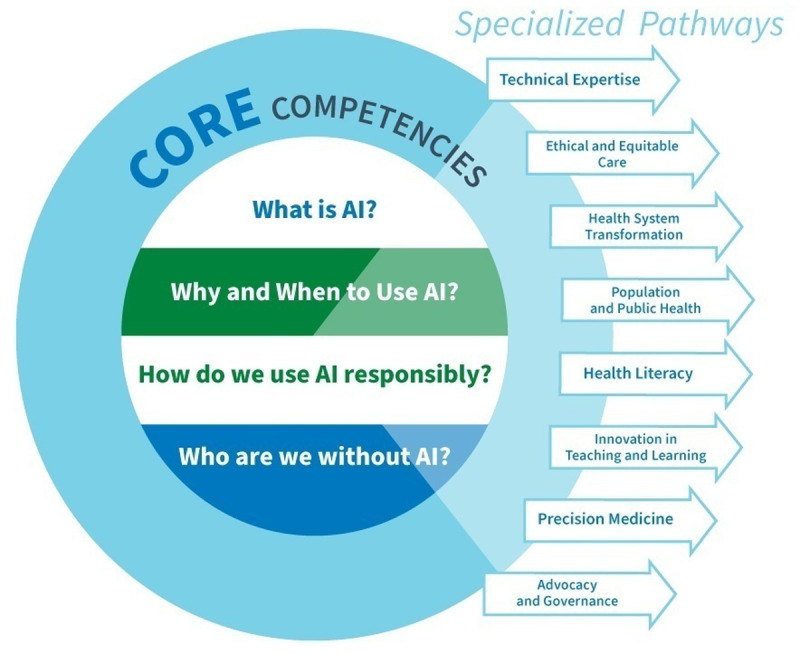
Graphic representation of four AI core competencies and eight specialized pathways.

We propose a set of foundational skills for all future physicians; with optional specialized pathways they can elect to pursue (Figure 1).

## Foundational Skills for AI Integration

To effectively practice in an AI-integrated healthcare environment, all physicians must acquire core skills. As AI becomes deeply integrated into their workflows, physicians will need to use AI efficiently, safely, and ethically [13]. Medical schools will need to integrate these skills as foundational competencies in their curriculum, ensuring that every graduating student is prepared for future practice.

Recent scholarship has established essential policy, governance, and competency foundations for integrating AI into medical curricula [14,15]. Our framework builds upon these. It extends beyond defining learner competencies to operationalize them through longitudinal, role-specific educational pathways that align with professional identity formation and leadership development. Others emphasize institutional readiness, standards of transparency, equity, and accountability. Our approach situates these competencies within an adaptive curricular architecture. It scaffolds basic literacy toward specialized expertise and leadership across domains such as technical innovation, health-systems transformation, and ethical governance. This integration positions learners not only as responsible users of AI, but as architects of its ethical and effective application in medicine.

### What is AI?

#### AI Literacy: Building essential understanding and proficiency

A solid grasp of AI fundamentals empowers students to use these tools effectively and safely. Just as understanding Wi-Fi is necessary to navigate smartphones, clinicians must learn key concepts such as pretraining, embedding, and fine-tuning to use AI intelligently. This knowledge helps them recognize AI’s strengths and limitations, troubleshoot issues, and apply it appropriately. For example, understanding pretraining can explain why models may overlook new conditions, while knowledge of transformers sheds light on hallucinations or incorrect citations. Every future physician needs these essential AI skills to succeed in an evolving healthcare landscape.

### Why and When to Use AI?

#### Effective AI Partnerships: Developing cognitive capability for intelligent collaboration

Knowing when to use AI, and when to question AI-generated responses, are crucial skills for future physicians [16]. Generative AI, can mimic expert human responses, posing risks such as AI dependency — where students stop thinking critically, and Automation Bias — where they accept AI’s responses without question [17]. Some medical educators may consider banning AI from curricula, akin to avoiding point-of-care ultrasound for fear that students won’t learn to use a stethoscope. However, the role of medical schools is to prepare students for the future, not shield them from it. Instead of prohibition, AI should be integrated into curricula through learning activities that encourage critical appraisal and reflection [17].

Developing critical thinking skills enables students to evaluate AI outputs against foundational knowledge identifying potential biases, hallucinations, and errors. As students harness AI to enhance learning, clinical skills, and research, fostering higher-order cognitive abilities, such as skepticism, curiosity, and critical appraisal, becomes essential. Just as a calculator enhances mathematical accuracy and efficiency, students must still estimate solutions to catch errors. Educators should embed AI into basic science, clinical, and research curricula while promoting analytical and reflective learning.

### How do we use AI Responsibly?

#### Ethics and Humanism in AI: Navigating the ethical landscape of AI Integration

As AI becomes embedded into practice, clinicians must incorporate patient values into decision-making and align AI with human-centered care [18]. Medical decisions are deeply personal, influenced by a patient’s unique values, preferences, and circumstances. While AI can analyze data and suggest actions, it cannot grasp the nuanced ethical and emotional aspects of patient care. Physicians must interpret AI insights through a human values lens, balancing AI with compassion and empathy.

Beyond ethical and humanistic considerations, responsible AI use must also account for its environmental footprint. The energy demands of large data centers and model training contribute substantially to carbon emissions, underscoring the need for sustainable, energy-efficient AI infrastructure.

Training should reinforce this balance, using interactive, case-based workshops to help learners explore AI’s ethical challenges in real-world contexts [19]. The current “straddle” generation of healthcare professionals bridges foundational medical principles with technological progress. Their role is critical — to guide the next generation in harnessing AI as a tool to enhance, not replace, medicine’s core values.

Recognizing AI’s biases is essential, and educators must equip students with strategies to identify and mitigate them. A humanistic approach prevents reducing patients to mere data points. Educators must model empathy-based care, reinforcing that medical knowledge is just one aspect of becoming an exceptional physician.

### Who are we without AI?

#### Adaptability and Resilience: Cultivating competence beyond AI reliance

Future physicians need the skills to adapt and deliver high-quality care even without AI. While AI integration may enhance efficiency and decision-making, its fallibility must be recognized. AI systems can experience downtimes from technical failures, power disruptions, cyberattacks, and connectivity issues. Reliance on AI can lead to cognitive deskilling, where clinicians lose proficiency in core clinical reasoning and decision-making skills. Training should ensure competence in medical knowledge and critical thinking skills independent of AI. Simulations and case-based learning without AI will support the development of strong clinical intuition, diagnostic acumen, and problem-solving abilities for environments where AI is unavailable or unreliable. Medical education curricula should adopt a balanced approach to prepare their students for both AI-augmented and AI-independent practice.

Beyond core competencies, medical educators should create longitudinal electives or dual degree programs focused on AI. Academic medical centers must collaborate across disciplines — and with industry as needed — to offer pathways specifically tailored to prepare future healthcare leaders to address challenges in an AI-driven healthcare system (Table 1).

**Table 1:** Recommended Collaborative Disciplines for the Proposed Educational Pathways.


PATHWAY	DISCIPLINES

Technical Expertise	Computer Science, Data Science, Biomedical Informatics, Information Security

Bias Reduction	Bioethics, Philosophy, Arts, and Humanities

Health System Transformation	Business, Management, Healthcare Administration, Industrial Engineering

Public and Population Health	Public Health, Sociology

Health Literacy	Communication, Education

Teaching and Learning	Education, Cognitive Psychology

Precision Medicine	Genomic Medicine, Biomedical Engineering

Advocacy and Governance	Law, Public Policy, Economics, Environmental Science


Educators should consider collaborations with these disciplines or others to develop specific expertise-based educational pathways.

##### Technical Expertise

###### Advanced technological mastery to drive AI innovation

Historically, healthcare has been slow to adopt new technology, often depending on externally developed solutions that lack substantial input from experienced health professionals, which in turn may fall short of meeting healthcare goals [20,21,22]. To build upon LLMs, such as ChatGPT for use in healthcare, it is imperative for healthcare professionals to either collaborate with AI developers or create solutions using their own proprietary data and expertise [23]. These initiatives include fine-tuning algorithms with healthcare data, developing custom solutions to allow organizations to use the LLM algorithms while preserving data privacy and security, and creating agents to automate repetitive tasks and reduce unnecessary cognitive load [24].

Maximizing AI’s potential benefits requires taking crucial steps to develop reliable, trustworthy, and customized applications that are safely integrated into patient care [25]. Training in this pathway will enable healthcare professionals to innovate and implement AI effectively and safely in patient care, education, research, and daily workflows. Furthermore, these AI experts can contribute to AI education by teaching curricula that train students and faculty.

##### Ethical and Equitable Care

###### Integrating ethical considerations, humanism, and bias mitigation

While training in understanding and mitigating biases is essential for all physicians, some trainees should specialize in designing and developing AI systems that reduce bias and enhance equitable care for all patients. LLMs’ recommendations are based on probabilistic algorithms that may not incorporate the values of individuals or specific groups [26]. Trainees in this pathway would be equipped to tackle these challenges in several ways. They would understand which values are embedded in LLMs and whose perspectives are represented, recognize when shifting data requires model retraining, and identify how and when biases arise. They could help clinicians balance individual patient values with AI recommendations and develop clinical decision support tools that detect and flag potential biases or inequities. Physicians trained in both AI and bioethics can also advocate for balanced regulations that address risks, promote fairness, establish standards, and ensure sustainable AI practices — ultimately fostering public trust [27].

By promoting a commitment to fairness, trust, and patient-centered care, medical education can empower future clinicians to navigate the ethical landscape of AI while upholding principles of equity and humanism.

##### Health Systems Transformation

###### Streamlining the functioning of complex healthcare systems

Recognizing the importance of how healthcare is delivered, health system science (HSS) has emerged as the third pillar of medical education. By integrating topics such as interprofessional education (IPE), value-based care, quality improvement, and systems thinking, HSS attempts to address the problems inherent in delivering care in complex healthcare systems [28]. Despite these efforts, institutions often face many challenges [29]. AI offers a transformative opportunity to optimize care in complex systems by leveraging large-scale data to identify patterns, predict risks, and trigger proactive interventions by clinicians and administrators. Innovations such as ambient AI and the Internet of Things (IoT) will transform how healthcare teams interact by integrating data from patient sensors, nursing notes, and lab results to facilitate more timely and effective delivery of care. AI systems can provide early warnings for patients experiencing decreased movement or a concerning trend in vital signs, facilitating urgent intervention and improving patient outcomes [30,31,32]. In quality improvement and patient safety efforts, AI can detect subtle signals of system flaws — such as near-miss events or variations in care delivery — prompting rapid process refinements. Moreover, AI-driven predictive analytics can enhance scheduling, resource allocation, and documentation processes, potentially reducing administrative burdens and caregiver burnout [33].

By incorporating system optimization as a learning pathway particularly for those in later stages of training or in clinical practice, providers will be equipped to lead in environments where operational excellence is vital. Training in AI applications for clinical and administrative transformation will prepare healthcare providers to navigate the intersection of technology, healthcare, and health systems science, improving patient care and system-wide operations.

##### Population and Public Health

###### Leveraging AI for community and population well-being

Persistent disparities continue to negatively impact healthcare outcomes, largely driven by socioeconomic determinants such as income, literacy, geography, and access to healthcare [28]. Marginalized populations often experience worse health outcomes, exacerbated by misinformation, varying levels of health literacy, and the politicization of health policies. Addressing these inequities requires coordinated efforts among health professionals, policymakers, and educators to create an equitable public health system.

AI can assist public health officials by managing extensive datasets from electronic health records (EHRs), patient monitoring devices, and other sources, aiding in pattern recognition and risk prediction. Techniques such as predictive modeling and deep learning can identify high-risk subpopulations in need of targeted interventions, whether for chronic disease management or preventive care. By linking clinical and social data, such as housing conditions or access to nutritional resources, AI can uncover underlying drivers of poor health outcomes and guide more tailored community-level interventions.

AI can boost disease surveillance, improve forecasting of chronic disease trends, and evaluate large-scale preventive interventions, equipping public health officials with essential tools for swift and informed responses to emerging health crises.

An educational pathway that integrates AI in population and public health will train providers to utilize data-driven techniques to improve health outcomes and tackle systemic public health challenges with innovative approaches.

##### Health Literacy

###### Tailoring patient education for improved outcomes

Health literacy, defined as the ability to access, understand, and apply health information, is crucial for achieving better health outcomes [34]. Low literacy levels can hinder patients’ understanding of diseases, adherence to treatments, and engagement in preventive health behaviors. Health professionals must recognize patients’ literacy levels and provide clear, accessible educational resources tailored to individual needs. Enhancement of communication methods and the thoughtful design of educational content can improve disease management and quality of care [35]. Generative AI can play a pivotal role by creating personalized, easy-to-understand materials and offering evidence-based explanations in diverse formats and languages [36]. These advances ensure that accurate, up-to-date, and accessible health information is available to all.

Moreover, AI applications can be deployed across wide-spread geographic areas and used by interprofessional teams to provide trustworthy information and reliable health guidance and recommendations for populations. Enhancing patient-facing tools with AI and making them available to under-resourced communities can help address health literacy, improve access and quality of care, and provide equitable value-based care [36,37].

Beyond bridging literacy gaps, AI-powered tools such as virtual tutors and chatbots, can provide immediate medical information and evidence-based guidance, supporting informed decision-making [38]. These tools increase patient engagement through personalized responses to health-related inquiries, appointment scheduling, and medication adherence reminders. Additionally, interactions with customized AI tools encourage critical thinking and can help debunk misinformation [39,40].

Trainees in this pathway will utilize AI to address the health education needs of patients, communities, and the broader public.

##### Innovation in Teaching and Learning

###### Incorporating AI to enhance educational practices

Medical education faces many challenges that impact its effectiveness and adaptability. Traditional training is lengthy, expensive, and designed with time-based progression. While competency-based education and personalized learning are the recognized solutions, they are currently difficult to implement at scale. Additionally, large numbers of faculty teach the same content at multiple institutions, but students frequently supplement these learning activities with third-party online resources. Today’s learners prefer multimodal, technology-driven methods that traditional curricula often fail to accommodate.

AI offers transformative solutions to these challenges. It can support precision medical education through individualized assessments and personalized learning plans, ensuring students receive customized training and feedback tailored to their unique strengths and weaknesses [41,42,43,44]. It can help students achieve competency faster, reducing training time while improving learning outcomes [45,46].

Beyond individualized learning, AI can democratize education by standardizing content across institutions, reducing redundancy and cost. Schools can collaborate on AI-powered teaching resources, fostering consistent, high-quality education while lowering faculty workload. AI can also support educators in admissions, assessments and education research [47], and in streamlining administrative tasks. This will allow educators to focus on developing these AI-based education solutions, using active learning for deeper dives into foundational concepts and on more human-centered skills.

Graduates of this pathway will drive the development of AI-integrated learning models that enhance education quality, efficiency, and accessibility. By embracing AI-driven innovations, medical education can evolve to meet the needs of future clinicians while optimizing resources and improving educational outcomes.

##### Precision Medicine

###### Harnessing Big Data and AI to personalize patient care

Precision medicine seeks to move beyond traditional, population-based approaches by tailoring healthcare to individual patients. The untapped potential of large, complex, and rapidly growing datasets is unlocked by advanced analytic techniques using AI. Providing targeted, effective, and personalized care will require AI analysis of electronic health records [48], genomic data [49], data from sensors and wearable devices [50], the microbiome [51] and information about social and environmental factors.

Students in this pathway would develop expertise in using AI-driven methodologies for big data analysis to implement precision medicine ethically and effectively.

##### Advocacy and Governance

###### Equipping clinicians with skills for responsible AI and data governance

As AI technologies become increasingly embedded in healthcare, the urgency for robust advocacy and governance is paramount. Historically, the rapid implementation of new technologies without adequate oversight has led to unintended consequences. The U.S. government’s push for Electronic Health Record (EHR) adoption, for example, was driven by the promise of improved care quality and efficiency [52], yet despite significant investment, EHR systems have introduced challenges such as poor usability, physician burnout, and adverse patient outcomes [53,54]. To prevent similar issues with AI, healthcare professionals must play an active role in shaping policies and regulations that align AI use with the needs of patients and providers. The American College of Physicians’ 2024 policy position highlights the need for increased regulatory oversight, research, and education in AI for clinicians and healthcare systems [33].

Physicians, as frontline healthcare providers, possess unique insights into the practical implications of AI in clinical settings. Their involvement in advocacy is crucial to ensure that AI-driven innovations enhance patient care rather than introduce new unforeseen burdens or disparities. Clinicians should receive training to critically evaluate and draft AI policies, advocate for resilient and environmentally sustainable funding approaches, and contribute to the development of regulatory frameworks that prioritize transparency, accountability, ethical considerations, and ecologic responsibility.

In addition, physicians must be trained to lead and supervise interprofessional teams that incorporate AI tools into clinical practice, ensuring safe, equitable, and transparent use across care settings. Developing these leadership and oversight competencies will enable future clinicians to guide responsible AI integration and mentor colleagues in its effective use [55].

Physicians trained in AI governance and advocacy will be prepared to navigate, influence, and advocate for ethical AI implementation, ensuring healthcare remains consistent with its core mission of equitable, high-quality patient care.

## Best Practices for Implementation

Implementing these changes will not be easy but medical educators have a huge responsibility and a once-in-lifetime opportunity to get this right. Successfully designing and implementing these core AI competencies and specialized pathways into medical education requires educators and institutions to adopt scalable, equitable, and sustainable practices. Equitable implementation also depends on addressing the financial barriers to AI adoption. The high cost of premium AI tools and licensing fees can limit access for learners and faculty, underscoring the need for institutional and consortium-level purchasing models, open-source alternatives, and shared licensing agreements to promote affordability and sustainability. Each proposed pathway can be tailored to learners at different stages of professional development. For example, technical innovation and systems-transformation tracks can be embedded into residency and fellowship programs, while leadership, governance, and executive education modules may suit practicing clinicians. Table 2 highlights representative models already implemented in UME, GME, and CME, demonstrating how AI leadership training can span the full continuum of medical education.

**Table 2:** AI in healthcare leadership pathways in UME, GME, and CME.


STAGE OF TRAINING	TRAINING TYPE	EXAMPLES

**For Medical Students**	Dual Degree Programs	University of Texas at San Antonio & UT Health San Antonio MD/MS in Artificial Intelligence (Direct 5-year program)

		Cleveland Clinic Lerner College of Medicine of Case Western Reserve University School of Medicine (CWRU SOM) 5-year program where most students graduate with a master’s degree (some in Biomedical and Health Informatics)

	Longitudinal Electives/pathways	University of Florida Artificial Intelligence in Medicine Research and Discovery Pathways Program

		CWRU SOM Medical Education Scholars Pathway, Advocacy and Public Health Pathway

		CWRU SOM – Healthcare Data Science with R and Python elective

**For Residents and Fellows**	American Board of Internal Medicine Research Pathway	Designed for traditional biomedical research, it could be adapted to conduct research on AI in Health

	PhD Tracks (For MD/PhD students or for graduate students)	Harvard Medical School Artificial Intelligence in Medicine PhD program

	Institution Specific Pathways	University of California, San Francisco (UCSF) – Clinical Informatics, Data Science, AI Pathway

		Mass General Brigham Data Science Pathway (Radiology)

	Master’s Programs in Data Science and Artificial Intelligence	University of Rochester online master’s program in Data Science and AI – designed for working professionals (including GME)

**For Practicing Clinicians**	Executive Education Certificate Programs (Some of the programs listed above for medical students and residents and fellows may also be available for practicing clinicians).	Harvard Medical School – AI in Health Care: From Strategies to Implementation – 8-week Online Instructor-paced course

		Stanford’s AI in Healthcare Specialization online 1-month self-paced course on Coursera

	Accredited Certification Programs	American Medical Informatics Association (AMIA) Health Informatics Certification (AHIC) Program. While targeted for health informatics, this model could be applied to AI certification programs.


Examples of adaptable models of educational pathways and programs preparing leaders in healthcare and AI across the continuum of education.

Proposed strategies include the following:

1. *Interdisciplinary Collaboration:*
  Designing and implementing curricula for these core competencies and pathways will require ongoing interdisciplinary collaboration (Table 1) and interprofessional teamwork between clinicians, educators, learners, and policymakers to ensure comprehensive AI integration. Effective implementation will also require close collaboration with information technology professionals, data scientists, and AI engineers who can help design, maintain, and optimize the digital infrastructure that underpins AI education and practice. They will help establish robust data infrastructures such as educational data warehouses and secure data pipelines to support AI-enabled learning, assessment, and research.
2. *Systems Thinking:*
  Educators and students need a holistic approach understanding the relationships and interdependencies in the complex healthcare system. Pathways should be designed, not to function in isolation, but with a focus on how each pathway interacts with the larger whole.
3. *Faculty Development:*
  Successful curriculum implementation requires faculty who are well-versed in AI concepts and applications. Institutions must ensure continuous professional development to keep educators updated in this rapidly changing field.
4. *Assessment and Evaluation:*
  Educators must develop robust metrics to assess learners and evaluate each pathway to ensure that the training efforts are producing competent physician leaders in each AI specialty area.
5. *Lifelong Learning:*
  To keep up with the rapidly changing landscape of AI, a dynamic, modular curriculum will be required to ensure graduates of these pathways can continuously update their AI knowledge as technology evolves.
6. *Global Perspectives:*
  The pathways should be designed to address global healthcare challenges. Educators and institutions should promote international collaboration to develop these curricula and share strategies for AI implementation.

## Embracing the Opportunity

The arrival of AI in healthcare heralds a transformative era, one that presents an extraordinary opportunity to reshape healthcare systems for the betterment of society. While AI challenges the current cognitive role of physicians, we should not fear being disrupted or replaced but instead lean in to leading the change to transform healthcare. By integrating AI into the continuum of medical education, we can address systemic challenges, improve patient outcomes, and empower healthcare professionals to lead in an AI-integrated future.

In the midst of unprecedented change, medical educators must seize this opportunity to re-envision the roles of healthcare professionals. AI can simplify healthcare complexities, making care more accessible and efficient, but unlocking its full potential requires cultivating new skill sets and fostering systems thinking. Future physicians must be equipped to navigate this evolving landscape with confidence and competence.

By embracing AI as a transformative ally, we can turn disruption into innovation. This moment demands bold, cross-disciplinary collaboration and a willingness to reimagine medical education to anticipate and shape the future of healthcare. If we fail to act, others will shape this future without us. Now is the time to harness AI’s disruptive potential to drive healthcare innovation and education, ensuring we meet the pressing healthcare needs of our patients and our world.

The authors, representing diverse perspectives, share a united belief in the power of bold, collaborative thinking. Together, we can reshape healthcare to serve society more effectively. As educators and leaders, we must prepare learners to embrace AI as a catalyst for positive change, ensuring that disruptions lead to meaningful and much-needed innovations. The future of healthcare is being written now — let us be the architects of its most promising chapter.
